# Is South Africa closing the health gaps between districts? Monitoring progress towards universal health service coverage with routine facility data

**DOI:** 10.1186/s12913-021-06171-3

**Published:** 2021-09-13

**Authors:** Candy Day, Andy Gray, Annibale Cois, Noluthando Ndlovu, Naomi Massyn, Ties Boerma

**Affiliations:** 1 Health Systems Trust, Durban, South Africa; 2 Discipline of Pharmaceutical Sciences, University of KwaZulu-Natal, Durban, South Africa; 3 Division of Epidemiology & Biostatistics, School of Public Health and Family Medicine, University of Cape Town, Cape Town, South Africa; 4 Department of Community Health Sciences, University of Manitoba, Winnipeg, Canada

**Keywords:** Universal health coverage, Service coverage index, Routine data, Survey data, Subnational disaggregation

## Abstract

**Background:**

South Africa is committed to advancing universal health coverage (UHC). The usefulness and potential of using routine health facility data for monitoring progress towards UHC, in the form of the 16-tracer WHO service coverage index (SCI), was assessed.

**Methods:**

Alternative approaches to calculating the WHO SCI from routine data, allowing for disaggregation to district level, were explored. Data extraction, coding, transformation and modelling processes were applied to generate time series for these alternatives. Equity was assessed using socio-economic quintiles by district.

**Results:**

The UHC SCI at a national level was 46.1 in 2007–2008 and 56.9 in 2016–2017. Only for the latter period, could the index be calculated for all indicators at a district level. Alternative indicators were formulated for 9 of 16 tracers in the index. Routine or repeated survey data could be used for 14 tracers. Apart from the NCD indicators, a gradient of poorer performance in the most deprived districts was evident in 2016–2017.

**Conclusions:**

It is possible to construct the UHC SCI for South Africa from predominantly routine data sources. Overall, there is evidence from district level data of a trend towards reduced inequity in relation to specific categories (notably RMNCH). Progress towards UHC has the potential to overcome fragmentation and enable harmonisation and interoperability of information systems. Private sector reporting of data into routine information systems should be encouraged.

**Supplementary Information:**

The online version contains supplementary material available at 10.1186/s12913-021-06171-3.

## Background

Achieving universal health coverage (UHC) requires the whole population to receive essential effective health services, without financial hardship. South Africa’s health system is fragmented between a relatively well-resourced private sector and an underfunded public sector, the latter responsible for the healthcare needs of the majority of the population. In 2012, the Lancet South Africa team noted marked improvements, but also the need to ensure “transformation of the health system into a national institution that is based on equity and merit” [1]. South Africa aims to advance towards UHC through the introduction of National Health Insurance (NHI) [2–4]. Effective governance of the health system relies on access to high-quality data on burden of disease, delivery of healthcare services, outcomes of interventions, and crucially, on the degree to which health equity is being advanced.

Global efforts to monitor progress in relation to the health-related target on universal health coverage, as part of the Sustainable Development Goal (SDG 3.8), have largely relied on survey data for between-country comparisons, in the form of the UHC service coverage index (SCI) [5, 6]. Alternative approaches have been reported, which combine service coverage and financial protection [7, 8]. Where direct measures of service delivery are not possible, proxy measures have been suggested, such as measures of capacity or health outcomes that are correlated with the provision of services [9]. To be more useful for regular subnational monitoring, alternative indicators based on routine or health facility data will need to be considered [10]. There is a need to monitor progress at the local (district, or even sub-district) level, to track implementation more frequently and to incorporate a quality of care component in the coverage measures where feasible.

South Africa has a mature system of monitoring district performance with health facility-based indicators. The Health Systems Trust (HST), a non-government organization working closely with the Department of Health, has published annual reports on the public sector in the District Health Barometer (DHB) since 2005. Drawing on a wider range of sources, the South African Health Review (SAHR) has included a chapter on Health and Related Indicators since 1995, focused on the national and provincial levels [11, 12]. The most recent issues of the DHB and SAHR report on the global UHC SCI*,* adapted to the country context [13–15].

This analysis assessed the usefulness and potential of routine health facility data for monitoring progress towards UHC. We focused on health service coverage (SDG 3.8.1) and not financial protection (SDG 3.8.2).

## Data and methods

We used the globally recommended UHC SCI as a starting point, and assessed the extent to which South African routine data could be used to calculate each of the 16 proposed indicators (referred to here as UHC1 to UHC16) at subnational level (provincial and district) [15]. We compiled data from 1998 to 2019, and constructed the index for selected time periods between 2007–08 and 2016–17 with sufficient data available for indicators in most categories, per district, province and nationally.

Indicators were updated to reflect the latest facility-collected data with consistent indicator formulations and time series population denominators aligned to the 2016 district boundaries. The UHC SCI was calculated as the geometric mean of the indicators by category after transformation and rescaling according to the guidance provided by the World Health Organization (WHO) [5, 9]. Where subnational data were not available for certain indicators (including ART effective coverage and Environment Health Services compliance rate) in earlier time periods, these indicators were excluded from the geometric mean calculation, not assumed to be zero or imputed.

Routine data for the public sector were collated from multiple sources, including the District Health Information System (DHIS), the financial management and human resources systems. The DHIS predominantly records and compiles routine data generated at facility level, and also includes exports from the disease-specific electronic registries established for the HIV and tuberculosis control programmes (Tier.Net). The reporting in the DHIS is informed by the National Indicator Data Set (NIDS) [16], which is periodically updated by the National Health Information System of South Africa (NHISSA) committee. In the private sector, the emphasis of routine data collection is on billing processes [17], and the data used were obtained from annual reports of the Council for Medical Schemes [18].

Survey data were used to validate health facility data where possible and to provide additional information to compute components of the index, as show in Table 1. The national Censuses and several surveys were considered, including the intercensal Community Surveys, and the annual General Household Surveys conducted by the national statistical service (Statistics South Africa (Stats SA)) [19]; the South African Demographic and Health Surveys (SADHS) [20]; the South African National Health and Nutrition Examination Survey (SANHANES) [21]; the South African National HIV Prevalence, Incidence, Behaviour and Communication Surveys (SABSSM) [22], and the annual National Antenatal HIV Prevalence Surveys [23]. Data from the first 5 ‘waves’ of a longitudinal panel study, the National Income Dynamics Study (NiDS), were also included [24].

**Table 1:** Country indicators and data sources for subnational UHC index

Tracer Area	Index indicator (WHO)	Index indicator, definition and transformations (South Africa)	Source
**Reproductive, maternal, newborn and child health (RMNCH)**			
UHC1: Family planning	Demand satisfied with modern methods in women aged 15–49 years who are married or in a union (%)	Couple year protection rate Women protected against pregnancy by using modern contraceptive methods, including sterilisation, as a proportion of female population 15–49 years.	DHIS
UHC2: Pregnancy and delivery care	Four or more visits to antenatal care (ANC) (%)	Antenatal 1st visit coverage before 20 weeks Number of ANC 1st visits before 20 weeks of gestation divided by the estimated number of pregnant women, for which the proxy is the population under 1 year multiplied by a factor of 1.15.	DHIS
UHC3: Child immunisation	Children aged 1 year who have received three doses of a diphtheria, tetanus, and pertussis (DTP) vaccine (%)	Immunisation under 1 year coverage Children under 1 year who completed their primary course of immunisation as a proportion of population under 1 year.	DHIS
UHC4: Child treatment	Care-seeking behaviour for children with suspected pneumonia (%)	Pneumonia case fatality under 5 years rate (smoothed and rescaled) Pneumonia deaths in children under 5 years as a proportion of pneumonia separations (inpatient deaths + inpatient discharges + inpatient transfers out) in health facilities. Smoothed rates rescaled according to the maximum observed value: $index=\frac{maximum- original\ value}{maximum- minimum} \bullet 100$	DHIS (modelled)
**Infectious diseases**			
UHC5: Tuberculosis (TB) treatment	TB effective treatment coverage (%)	TB effective treatment coverage Treatment success (drug-sensitive) multiplied by the national WHO estimate of the case detection rate.	DHIS-Tier.net (electronic HIV and TB register data as exported to DHIS)
UHC6: HIV treatment	People living with HIV (PLHIV) receiving antiretroviral therapy (%)	Antiretroviral effective coverage Total number of PLHIV, on treatment, with viral load suppressed at 12 months divided by the total number of PLHIV.	DHIS-Tier.net PLHIV estimates were derived by a modelling and triangulation method, incorporated in DHIS.
UHC8: Water and sanitation	Households with access to at least basic sanitation (%)	Percentage of households with access to improved sanitation Proportion of households with access to flush toilets connected to a public sewerage system or a septic tank, or to a pit toilet with a ventilation pipe.	Census and Community Survey
**Non-communicable diseases (NCD)**			
UHC9: Prevention of cardio-vascular disease	Prevalence of non-raised blood pressure regardless of treatment status (%)	Age-standardised prevalence of non-raised blood pressure (rescaled) Proportion of population 15 years and older with systolic blood pressure < 140 mmHg and diastolic blood pressure < 90 mmHg. Age-standardised using population estimates from Census 2011 and rescaled to obtain finer resolution across districts: $index=\frac{original\ value- minimum}{100- minimum}\bullet 100$	NiDS
UHC10: Management of diabetes	Age-standardized mean fasting plasma glucose for adults aged 25 years and older	Diabetes treatment coverage Ratio between the population proportion of treated cases and diabetes prevalence.	NiDS, modelled using predictor information of diabetes prevalence from SADHS
UHC11: Cancer detection	Cervical cancer screening in women aged 30–49 years (%)	Cervical cancer screening coverage Cervical smears in women 30 years and older as a proportion of one-tenth of the female population 30 years and older.	DHIS
UHC12: Tobacco control	Adults aged at least 15 years who had not smoked tobacco in the previous 30 days (%)	Tobacco non-smoking prevalence Adults aged at least 15 years who had not smoked tobacco in the previous 30 days.	NiDS
**Service capacity and access**			
UHC13: Facility access	Number of hospital beds per person	Hospital beds per 10,000 target population (rescaled) Total number of public sector hospital beds per 10,000 uninsured population, rescaled using the target of 18 beds per 10,000 population. $index=\frac{original\ value}{18}\bullet 100$	DHIS Modelled estimates of the uninsured population from Census, Community Survey, General Household Survey and Council for Medical Schemes data.
UHC14: Health worker density	Number of health professionals per person: comprising physicians, psychiatrists, and surgeons	Health worker density (rescaled) Geometric mean of the scaled scores for medical practitioners (mp), professional nurses (pn) and pharmacists (ph) employed in the public sector, using thresholds of 30 physicians, 100 nurses, and 5 pharmacists per 10,000 uninsured population. $index=\sqrt[3]{\frac{mp}{30}\bullet \frac{pn}{100}\bullet \frac{ph}{5}}\bullet 100$	PERSAL Modelled estimates of the uninsured population.
UHC15: Access to essential medicines	Proportion of health facilities with availability of the WHO-recommended core list of essential medicines	Proportion of primary health facilities with essential medicines 100 minus the tracer items stock-out rate in fixed primary care facilities.	DHIS
UHC16: Health security	International Health Regulations core capacity index	Environmental health services compliance rate Based on national environmental health norms and standards which municipalities should adhere to, assessed by means of an audit tool.	National Department of Health audit tool

Multiple modifications from the WHO UHC SCI were needed to adapt the construction to local availability of data. The index indicators, definitions, transformations and sources applied for the subnational South African index are shown in Table 1. Alternative indicators were formulated for 9 of 16 tracers in the index. Routine or repeated survey data could be used for 14 tracers. The malaria indicator (UHC7) was excluded, as insecticide-treated bednets are not routinely provided in South Africa.

UHC1 was based on the couple year protection rate (CYPR), which uses service delivery data on individual contraceptive methods supplied [16]. The time series was updated to take into account a change in the weights used for male and female condoms [25]. The increase in CYPR may be the result of the considerable increase in male (and to a lesser extent female) condom distribution in the context of the AIDS epidemic. CYPR cannot distinguish single from dual method use, so may over-estimate the proportion of couples protected. By 2016, there was remarkable correspondence between the DHIS and SADHS data, even though the survey measures both demand and supply, and the routine measure is focused only on supply (see Figure A1 in Additional file 1).

Instead of the percentage of births attended by skilled health personnel for UHC2, WHO suggests tracking the percentage of ANC clients with first visits before 12 weeks [26]. UHC2 was instead calculated from DHIS elements as the proportion of pregnant women who attend an antenatal clinic before 20 weeks’ gestation to incorporate both coverage and a measure of quality (timely care).

UHC3 was based on the percentage of those under 1 year who have received all vaccines in the national regime, in line with the global trend towards tracking multiple antigens [7]. In South Africa, the composite immunisation coverage measure has the longest and most stable time series of data collected routinely.

The SADHS series has provided only a single 2016 national estimate of childhood pneumonia treatment [20]. UHC4 was instead based on rescaled smoothed estimates of the pneumonia case fatality rate (CFR) under 5 years. The maximum observed CFR varied considerably over time, so caution must be exercised when interpreting the rescaled value. The smoothing procedure is described in Additional file 1.

UHC 5 is about effective coverage of TB treatment. Outcomes data always lag the current reporting period by one or two years, as the cohorts are constructed from a common treatment initiation time point [27]. Construction of the effective coverage index measure requires consideration of the case notification rate (the proportion of all people who have TB who are screened, diagnosed and initiated on treatment). The single national estimate based on expert opinion was used for this index [28].

Subnational numbers of PLHIV are needed to calculate UHC6 on antiretroviral treatment. A time series of district level estimates for children (0–14 years) and adults (15 years and older), based on triangulation of three HIV-prevalence data sources was updated and used [29]. Viral suppression outcomes enabled assessment of effective coverage. South Africa’s HIV epidemic model, Thembisa, provides longer time series of HIV-related data, including private sector contributions, but does not allow for disaggregation to district level [30, 31]. ART effective coverage reported from Thembisa is considerably higher than that recorded in DHIS-Tier.net. Dwyer-Lindgren et al. have pointed out how the bespoke Thembisa model differs from that used by other countries [32].

UHC9 was calculated from the rescaled age-standardised prevalence of non-raised blood pressure in the adult population, regardless of treatment status. Although multiple surveys have reported biomarkers for blood pressure, the 5 ‘waves’ of NiDS provide the most frequent measures and are the only survey sources which can be disaggregated to district level. Alternative indicators are also feasible, including treatment coverage and effective treatment coverage of hypertension (Additional file 1) [33]. Although the level of coverage varies substantially according to the choice of index indicator (from 16.4% for effective treatment coverage to 79.3% for the age-standardised prevalence of non-raised blood pressure), all results suggest a similar trend of improved coverage. Cois et al. inferred that, despite worsening risk factors for elevated blood pressure, expanded antihypertensive treatment contributed substantially to the downward trend in uncontrolled hypertension [34].

Only a single national figure for diabetes treatment coverage (UHC10) has been reported in SANHANES 2012 [35]. No published reports of hypertension or diabetes treatment coverage using health facility data were found, and the utility of current indicators is limited [33, 36]. Meanwhile, indirect estimates of treatment coverage for diabetes were obtained by modelling. Details on the procedure are provided in Additional file 1. A machine learning algorithm was trained with data from SADHS 2016, which includes biomarkers for direct estimation of diabetes status, to predict individual probabilities of being diabetic from demographic (age, sex, ethnicity) and bio-behavioural (body mass index, waist circumference, current smoking) characteristics and self-reported diagnosis and medication use. The model was then applied to data from the NiDS survey (which does not include biomarkers) to estimate, for each ‘wave’, prevalence of diabetes at national and subnational level, while the proportion of treated subjects was directly estimated from self-reported data. Treatment coverage was then calculated as the ratio between the population proportion of treated cases and diabetes prevalence. A smooth variation over time was assumed for treatment coverage, and final annual estimates were obtained by thin-plate spline smoothing.

UHC11 could be obtained from routine data. Survey data, such as those in SADHS, are reported for women who have ever had a cervical cancer screening test, how long ago that was, and whether they got the result. Comparing routine and survey results is therefore complicated, with many possible reasons for differences. The current routine indicators are constructed using one-tenth of the female population 30–49 years for the denominator (since women are recommended to be screened once in 10 years). Aggregated data do not distinguish between multiple tests in the same woman and unique encounters. This may be one reason why the routine indicator is almost twice as high (63.6%) as that reported in SADHS (36.5%) in 2016. Cervical screening is however recommended more frequently, from age 20 in those living with HIV, resulting in further over-estimation of coverage. A new formula for the calculation of the routine indicator is being implemented, taking HIV prevalence into account.

UHC12 could only be determined from surveys, since no routine data on smoking are collected, nor are there plans to do so.

The hospital bed density indicator (UHC13) was calculated as the rescaled number of beds in public hospitals per 10,000 uninsured population.

For the health worker density index (UHC14), the specialist subcategories included in the global index defined by WHO could not be distinguished. An index based solely on medical practitioners is not meaningful in the context of the South African health system where nurses form the backbone. The index was calculated according to the methodology of the GBD 2017 SDG Collaborators [7] as the geometric mean of the scaled scores for each of three key professions (medical practitioners, professional nurses, pharmacists), with reference to the uninsured population. Public sector data were extracted from the Personnel Administration System (PERSAL) and subjected to extensive data coding to identify occupational classifications and geographic location, as personnel data are not routinely reported by district.

The choice of restricting the calculation of the previous indicators to the uninsured population was dictated by the lack of comprehensive data on hospital beds and health personnel for the private sector. The proportion of population not covered by medical insurance was estimated by Insight Actuaries [37] using a small area model and multiple data sources. Details on the procedure are provided in Additional file 1.

The International Health Regulations (IHR) core capacity index (UHC16) is only reported at a national level. The environmental health services compliance rate was used instead [38].

Subnational indicators were subjected to an equity analysis, using socio-economic quintiles derived from the South African Index of Multiple Deprivation (SAIMD) developed by Noble et al. and adapted for the District Health Barometer [39, 40].

## Results

Using routine data where possible, the value of the UHC SCI could be fully derived for 2016–17, and partially (i.e. with the omission of some of the indicators for lack of data) for the years 2007–2008, 2012, and 2015. Although routine systems contain data for selected indicators from as early as 2000, there were insufficient data in some categories to calculate a useful UHC SCI for earlier years and, similarly, for more recent years.

**Fig. 1:**
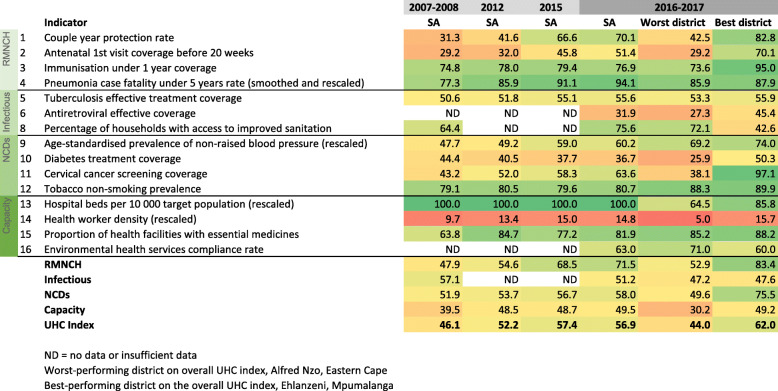
Trends in the South African UHC SCI

**Fig. 2:**
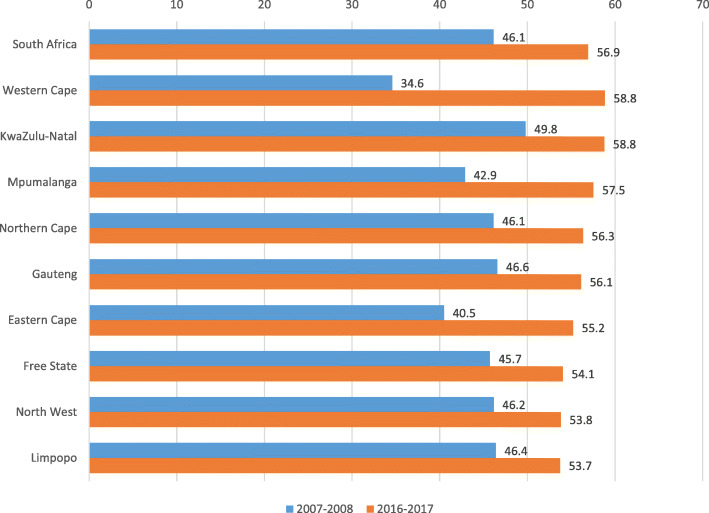
UHC SCI at national and provincial level between 2007 and 08 and 2016–17

The national values of the index are shown in Fig. 1, together with the 2016–2017 values for the best and worst performing districts, on the overall UHC SCI, Ehlanzeni (DC32, Mpumalanga province) and Alfred Nzo (DC44, Eastern Cape province). The values for the best and worst districts are for DC32 and DC44 and do not represent the highest and lowest values in any district. Figure 2 compares the results at provincial level.

The UHC service index improved considerably in South Africa as a whole and in all provinces during the period between 2007−08 and 2016–17. The national index increased from 46.1 to 56.9. Among the four categories, the greatest progress was made in the RMNCH category, which increased by 49%, from 47.9 to 71.5. The apparent decrease in the infectious disease category between 2007−08 and 2016–17 is an artefact, as the earlier index was calculated on only two indicators due to the non-availability of ART effective coverage data at district level. Including the low ART effective coverage value in 2016–17 resulted in a 10% decrease in the infectious disease category, despite improvement in the tuberculosis and sanitation indicators.

Details on the specific indicators are shown in Additional file Table A3. All provinces made progress but major gaps remained. The greatest increase in absolute value of the UHC SCI was made in the Western Cape, Eastern Cape and Mpumalanga provinces, although the increase in Western Cape was partly driven by a low value for access to essential medicines in the first period (data quality issues). The smallest improvement in the index was in Limpopo. By 2016–17 there were still large gaps between the provinces, with 5.1 points difference between the best performing (KwaZulu-Natal and Western Cape, with a score of 58.8) and the worst performing province (Limpopo, with a score of 53.7). Although substantial progress was made in most districts, the worst performing district in 2016–2017, Alfred Nzo (Eastern Cape), only increased by 3 points to 44.

**Fig. 3:**
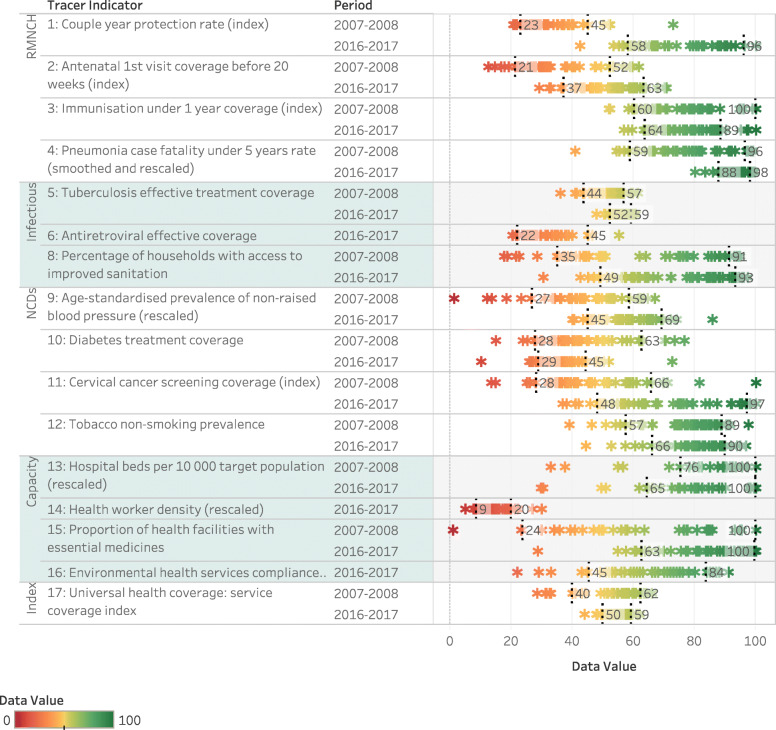
Trends in UHC tracer indicators range by district showing 10–90 percentile range

The distribution of UHC tracer indicators and the summary index by district shows progress for many indicators (Fig. 3). With a few exceptions, the median value across districts improved with a clear tendency toward a reduction in range. The differences, however, remain large, with a 10–90 percentile range of 9.3 overall, but as high as 48.7 for cervical cancer screening coverage.

Consistent improvement is evident in relation to the RMNCH indicators. Reduced inequity in TB outcomes is apparent although treatment success in the best-performing districts has stagnated. In relation to the NCD indicators, progress has been noted in non-raised blood pressure and cervical cancer screening. Diabetes control poses far more challenges. Little change in the distribution of hospital bed density can be discerned. Although the short time series for district-level health worker density does not show change over time, this coincides with a period of economic austerity. Aggregated to the national level, the density has almost doubled since 2001.

**Fig. 4:**
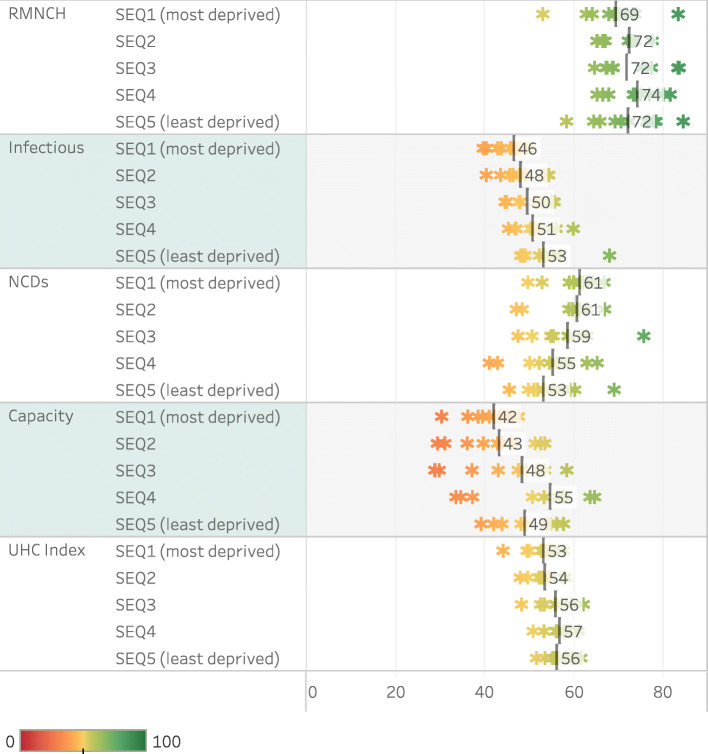
UHC SCI by socio-economic quintile of districts, range and median, 2016–2017

The spread of index categories and the final index for 2016–2017 between districts of different socio-economic quintiles is shown in Fig. 4, where SEQ1 is the most deprived and SEQ5 is the least deprived. There tends to be a gradient from poorer performance in deprived districts to better performance in less deprived districts, except in the NCD category. The somewhat low Capacity scores in SEQ5 (which includes most metros and the highest density of private facilities) may reflect the absence of private sector data. Frances Baard and Buffalo City districts in SEQ4 are outliers (high) as they provide higher level services to surrounding areas.

## Discussion

We have shown that it is feasible to use the WHO approach, with modifications, to assess progress towards UHC with an index largely based on health facility data. We showed that using health facility data from the public sector in South Africa it was possible to obtain insights into the different domains of UHC at subnational levels, and to some extent track progress over time. The national UHC SCI value of 56.9 for 2016–2017 is lower than that previously computed (66.2) [14] and the global estimate of 69 [6]. It is also slightly lower than the updated UHC SCI based primarily on survey-based data sources (61.8 for 2016–17) although some indicator or data source substitutions within the index lead to variation in opposite directions [41]. There was an improvement in the index across most indicators and geographic levels, which is consistent with the concerted efforts to strengthen the district health system. The absence of data for UHC6, UHC14 (district level) and UHC16 in 2007–08 needs to be noted. Nonetheless, effective ART coverage would have improved drastically over the time period. A lag in the availability of some indicators meant that the index could not be computed for the most recent time period. The 2016–2017 data were therefore the most recent complete set.

This UHC SCI differs in important respects from that reported by WHO, in order to maximise the use of routine data, and potentially show how South Africa has progressed in advancing equity [6]. The intent of the WHO UHC SCI was to allow for the measurement of country performance using available data [15], but also to refine the index as more nuanced measures of effective service coverage became available. Greater reliance on routine rather than periodic survey data should allow for more intensive tracking of progress. Disaggregation to subnational levels enables a more granular focus on equity. Collating routine data over an extended period poses challenges where there are missing data. Although there are sensitivities with using values that deviate from the raw data reported, the modelling exercise relied on to estimate pneumonia CFR per district has potential. Smoothed estimates are important where single-disease fatalities in one age group may be fairly rare and fluctuate dramatically, especially in districts with small populations and well-functioning health systems. More work is needed to validate whether this is a suitable proxy for child treatment as the impact of pneumococcal immunisation changes incidence patterns, hospitalisation and chronic morbidity following pneumonia [42, 43]. However, it has also been pointed out that cross-sectional surveys are not a reliable measure of the proportion of children with pneumonia accessing care, and cannot easily be disaggregated [44].

Despite multiple challenges, a comprehensive set of indicators for effective coverage of all sectors of the population was produced, over a time series, with disaggregation to current district boundaries, and by equity stratifiers. However, most routine health facility indicators exclude the private sector (serving around 15% of the population, but this varies widely across districts from 4 to 31%). The solutions proposed may assist South Africa and other countries to strengthen routine approaches to subnational monitoring of UHC.

Discrepancies between immunisation coverage based on surveys and routine data have been recognised in South Africa [45–47]. Estimates based on routine data have been bedevilled by underestimation of the population under 1 year of age, especially at subnational level [48–50]. Ongoing engagement with Stats SA to revise population projections has mitigated many of these challenges. The national vaccination coverage survey, in the field in 2019, should provide the first reliable vaccination coverage estimates at district level [51].

Addressing the lag in TB treatment outcomes reporting could be achieved by changing the cohort construct to one based on the treatment endpoint instead of treatment start date. This would allow for consolidated reporting of drug-sensitive and drug-resistant cases, whether treated with long or short courses. Calculating TB effective treatment coverage at sub-national level requires access to sub-national case notification rates rather than a single national estimate. Naidoo et al. detailed the losses at each step in the care cascade, concluding that just over half of the estimated cases were successfully treated in 2013, which is in line with the estimate relied on for this index [52]. Preliminary efforts to quantify the number of ‘missing’ TB cases per district have been reported but were not suitable for use in calculating this index [27, 53]. The first national TB prevalence survey will provide an alternative once released (now expected in 2021) [54].

The utility of the hospital bed density indicator (including all levels of care) is limited in South Africa, as the country appears to have achieved the target. Some inequity is, however, apparent at district level. It could be argued that the most sensitive measure for sub-national monitoring would be the number of district hospital beds per 10,000 uninsured population, but a new threshold would need to be determined. A possible alternative to the hospital bed count is the percentage of fixed primary care facilities that are assessed as ‘ideal’ (meeting defined quality standards) [55, 56]. Clinics in the public sector have only recently been assessed, not all facilities are assessed every year, and refinements to the assessment tool are still ongoing. More robust measures of quality service provision may become more useful in time, especially as the structured tools for assessment mature. Healthcare utilisation rates are a useful measure of the implementation of UHC plans [57]. The provision of healthcare facilities has been emphasised in a recent determination of the number of excess deaths in low- and middle-income countries amenable to personal healthcare services, and the portions attributable to non-utilisation of healthcare versus those attributable to receipt of poor-quality care [58]. Neethling et al. have restated the data from the 2nd South African Burden of Disease study in amenable mortality terms for 45 causes of death [59].

Routine laboratory data or electronic health records should be reliable sources of data on the proportion of patients on ART who are virally suppressed. However, a study conducted in Khayelitsha showed that while 84% of HIV viral load determinations were actually performed, only 79% had the results filed, 76% were noted and 55% were captured in the electronic health record maintained in Western Cape health facilities [60].

Potential adjustments can be explored where issues with data quality are identified. There are concerns that the improvement in CYPR has been driven predominantly by changes in condom distribution, or the recording thereof, and that CYPR figures are an over-estimate of the level of contraceptive cover [25]. Adjusting the CYPR formula could address this problem, but research and consultation is needed to evaluate what factors are realistic and how those might vary between country settings. The conversion factors proposed by Stover et al. in 2000, do not adequately address this issue [61].

A key challenge to comprehensive national monitoring remains the separation between the public and private sectors. Using proxy data (for example anthropometric data) from nationally-representative survey data to model the prevalence of a particular disease (in this case diabetes mellitus) can help bridge that divide. The COVID-19 pandemic has demonstrated how shared objectives can encourage joint reporting from both sectors, even without legislative action. An example is the DATCOV hospital surveillance system to track COVID-19 admissions in private and public hospitals [62]. The barriers to private sector reporting into DHIS are not insurmountable.

In order to track progress through an equity lens, districts are categorised by SEQ, based on the SAIMD [39]. In general, rural districts are more likely to be deprived than urban districts, and rural districts located in the former *apartheid*-era ‘homelands’ are likely to be more deprived than even peri-urban and informal settings [40]. The ranking of districts has not changed between the 2001 and 2011 Censuses. Apart from the NCD indicators, a gradient of poorer performance in the most deprived districts was evident. This emphasises the need to look at the categories within the index, and not only the summary figure. Although progress in some sub-Saharan African countries has been noted, large inequalities in RMNCH measures at subnational level persist [63].

Caution is needed in assessing progress over time when important indicators have missing data for some of the time series. A limitation of this analysis is that missing data were not imputed for all indicators. This is an important area for future work. Another important limitation is the lack of private sector data (around 15% of the population are privately insured), which will skew individual indicators differentially. Caution should also be exercised when comparing this modified index with that applied in other countries, where the modifications may not be comparable.

This analysis did not assess financial risk protection. Ideally both the service coverage dimension and financial risk protection should be combined in one summary index of UHC and progress towards SDG 3.8 [64].

## Conclusion

SA has made measurable progress towards UHC. There is evidence from district level data of a trend towards reduced inequity in relation to specific categories of the UHC SCI (notably RMNCH). The lack of human resources for health remains a persistent feature across provinces and districts. The development of a single payer system has the potential to overcome much of the fragmentation that has persisted, even 25 years after the democratic transition, and would also enable the harmonisation and interoperability that exists on paper, in policy documents, but not yet in reality. In the interim, encouraging private sector reporting of data into the DHIS is needed to address existing fragmentation. Increasingly, it is possible to construct the UHC SCI from predominantly routine data sources. Well-designed national surveys, with sufficient sample sizes to enable disaggregation to small areas, will remain important. Proxy data from multiple sources can enable new approaches to determining disease-specific prevalence estimates that are otherwise time consuming and expensive to measure directly. Co-ordination and planning of such surveys at suitable intervals and disaggregation are crucial. Additional analytical techniques will be needed to address the multiple limitations in measuring progress over time across all indicators for the whole population, as well as for vulnerable groups. The widespread use of DHIS in other low- and middle-income countries should enable the application of the approach described here. However, the challenges of measuring effective coverage of NCDs without regular sub-national prevalence surveys will need to be faced.

## Supplementary Information

**Additional file 1.** Methodological details and additional results.

## Data Availability

We used multiple sources of data as described in the paper, as obtained routinely for the production of the District Health Barometer published by the Health Systems Trust and accessible from www.hst.org.za.

## Abbreviations

CFR: Case fatality rate
DHB: District Health Barometer
DHIS: District Health Information System/Software
HST: Health Systems Trust
NHI: National Health Insurance
NHISSA: National Health Information System of South Africa
NiDS: National Income Dynamics Study
NIDS: National Indicator Data Set
PERSAL: Personnel Administration System
SABSSM: South African National HIV Prevalence, Incidence, Behaviour and Communication Survey
SADHS: South African Demographic and Health Survey
SAHR: South African Health Review
SAIMD: South African Index of Multiple Deprivation
SANHANES: National Health and Nutrition Examination Survey
SCI: Service Coverage Index
SDG: Sustainable Development Goal
SEQ: Socio-economic quintile
UHC: Universal Health Coverage
WHO: World Health Organization
