# Supplementary material for: Is South Africa closing the health gaps between districts? Monitoring progress towards universal health service coverage with routine facility data
Source: BMC Health Serv Res. 2021 Sep 13;21(Suppl 1):194. doi: 10.1186/s12913-021-06171-3 (PMC8435360; doi:10.1186/s12913-021-06171-3)
Supplement: Supplementary file 1 — Additional file 1. Methodological details and additional results. [file 12913_2021_6171_MOESM1_ESM.pdf]

Is South Africa closing the health gaps between districts? Monitoring progress towards universal health service coverage with routine facility data

Candy Day, Andy Gray, Annibale Cois, Noluthando Ndlovu, Naomi Massyn, Ties Boerma

Additional File 1: Methodological details & additional results

1. Methodological details 1
   1.1. Comparison of surveys vs. routine indicators for family planning 1
   1.2. Smoothed estimates of pneumonia deaths in children under 5 years as a proportion of pneumonia separations under 5 years in health facilities 2
   1.3. Comparison of alternative indicators and sources for prevention of cardiovascular disease 4
   1.4. Estimation of treatment coverage for diabetes 4
   1.5. Estimation of proportion of population not covered by medical insurance 6
2. Additional Results 7

1. Methodological details

1.1. Comparison of surveys vs. routine indicators for family planning

Figure A1: Comparison of survey and routine indicators for family planning

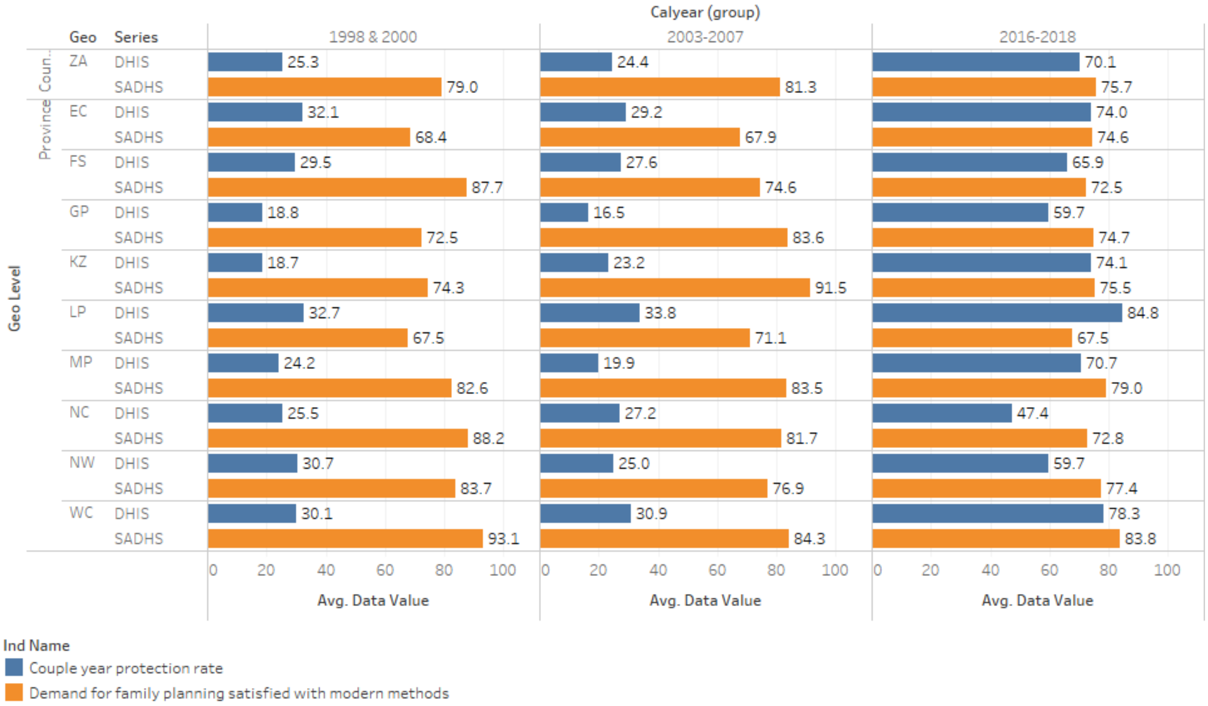

DHIS = District Health Information System
SADHS = South African Demographic and Health Survey
ZA = South Africa; EC = Eastern Cape; FS = Free State; GP = Gauteng Province; KZ = KwaZulu-Natal; LP = Limpopo Province; MP = Mpumalanga; NC = Northern Cape; NW = North West; WC = Western Cape

1.2. Smoothed estimates of pneumonia deaths in children under 5 years as a proportion of pneumonia separations under 5 years in health facilities

All available data on case fatality rate for pneumonia in children < 5 years were extracted from the DHIS for the period between 2005 and 2018. For each district, a 'raw' value for the indicator was calculated as the proportion of deaths over the total number of facility separations (defined as the sum of the number of deaths + the number of discharges + the number of transfers to other facilities) in the reference year.

Data were visually inspected for the presence of extreme outliers and implausible values, and as a result 4 values (> 10 times the average values for the district over the whole period) were excluded from the dataset.

A generalised additive log-log model with thin-plate splines was fit to the remaining values, separately for each district. The estimated model coefficients were used to generate a smoothed, consistent series of values for the indicator. The smoothed estimated are shown in Figure A2 (blue lines) together with the raw values (red squares).

Figure A2: Smoothed vs. raw estimates of case fatality ratio (CFR) for pneumonia in children < 5 years, by district

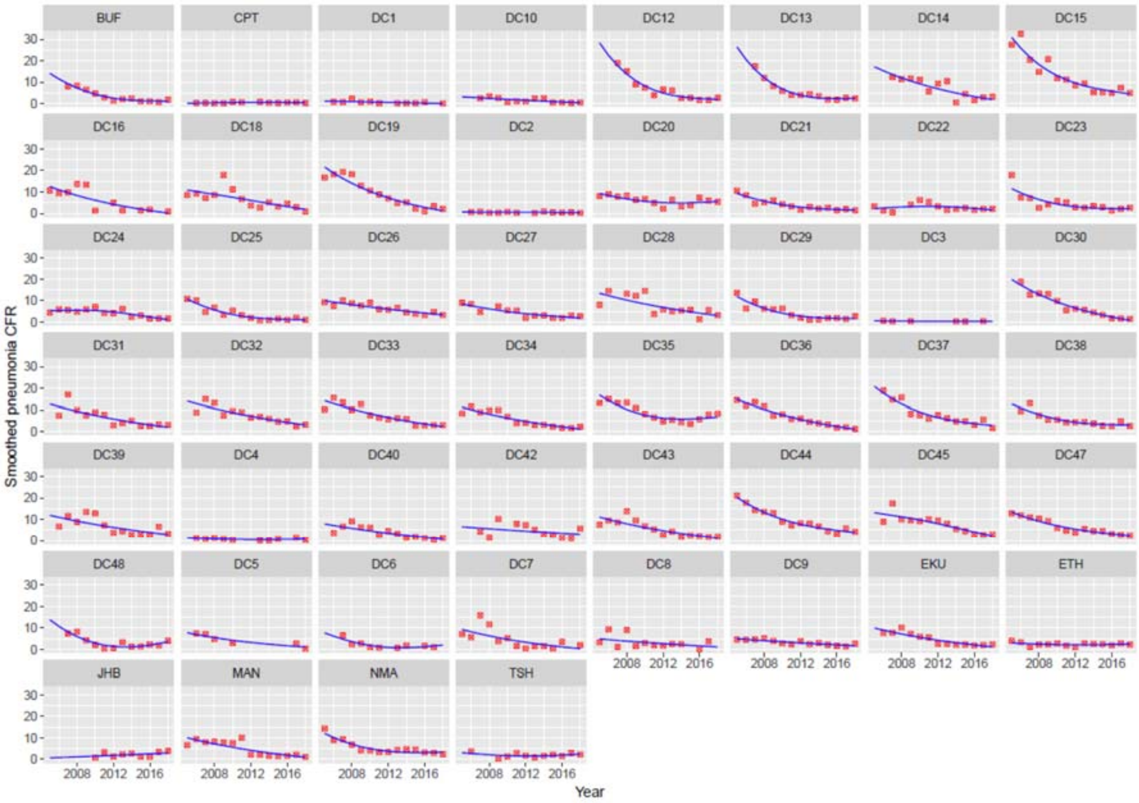

The smoothed estimates were rescaled according to the maximum observed value

$$index = \frac{maximum\ - original\ value}{maximum\ - minimum} \cdot 100$$

The results at district and province level are shown in Figure A3 and A4, respectively.

Figure A3: Smoothed vs. raw estimates of the UHC4 SCI indicator (Child treatment), by district

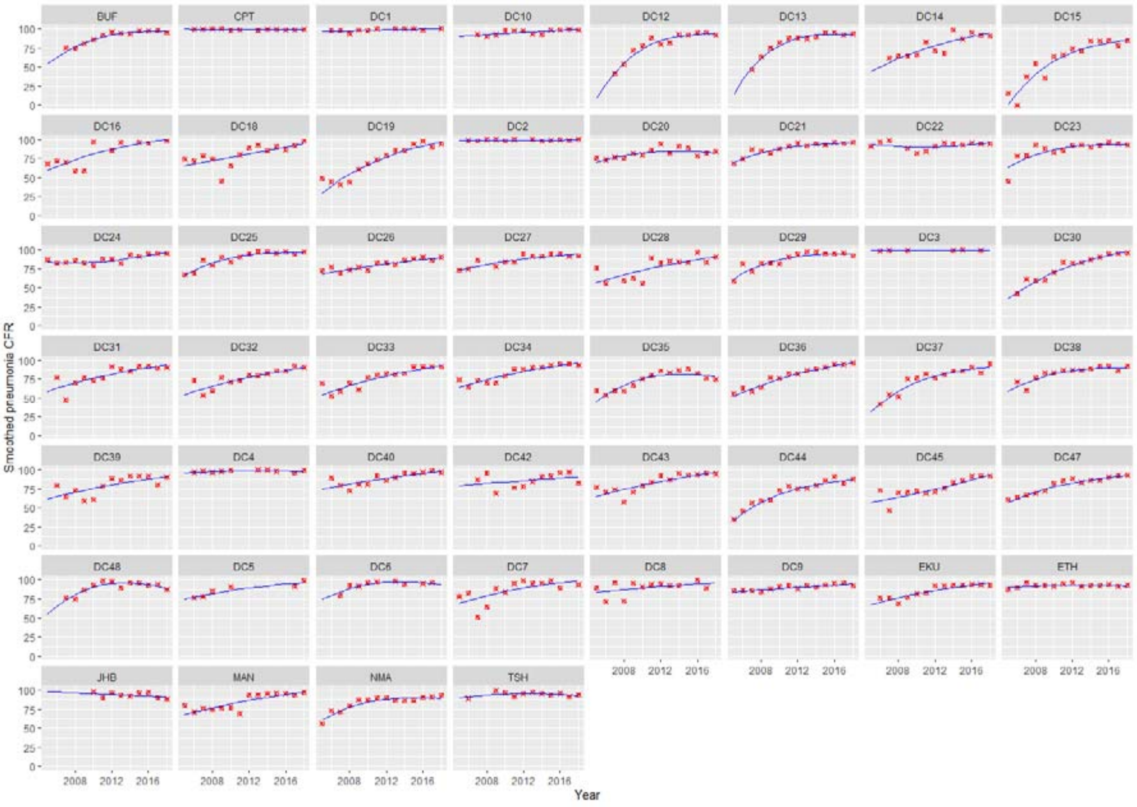

Figure A4: Smoothed vs. raw estimates of the UHC4 SCI indicator (Child treatment), by province

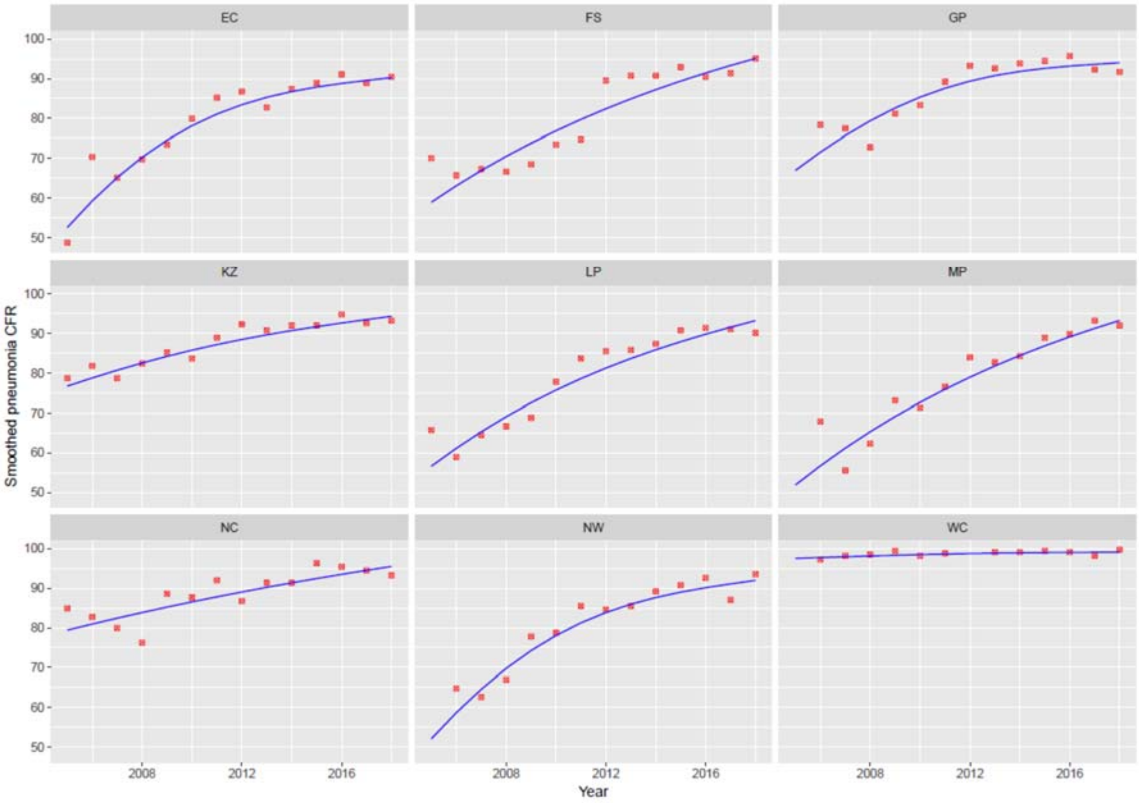

1.3. Comparison of alternative indicators and sources for prevention of cardiovascular disease

Figure A5: Comparison of alternative indicators and sources for prevention of cardiovascular disease, South Africa

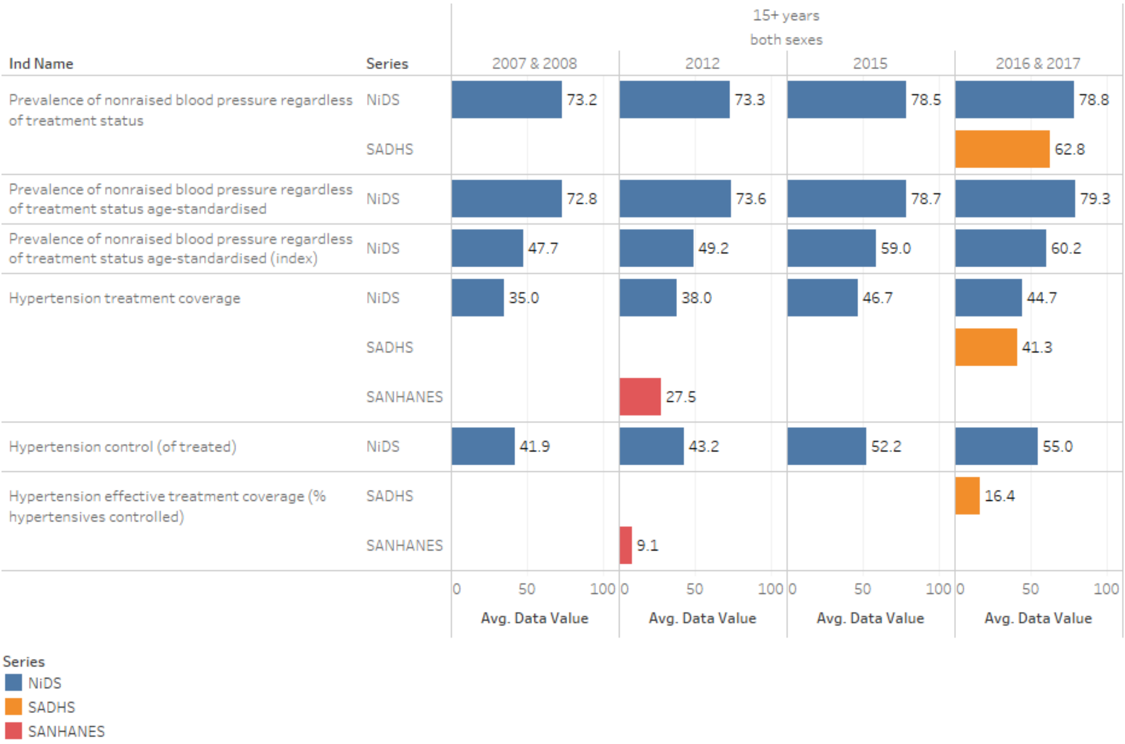

NiDS = National Income Dynamics Study
DHIS = District Health Information System
SADHS = South African Demographic and Health Survey

1.4. Estimation of treatment coverage for diabetes

Only a single national figure for diabetes treatment coverage has been reported for South Africa, in SANHANES 2012.[1] No published literature could be found that reported hypertension or diabetes treatment coverage indicators using health facility data, and the utility of current indicators is limited.[2, 3] Routine data on chronic disease visits and treatment initiations could potentially be combined with estimates of prevalence (from surveys) to fill this gap. The first review of all available studies on diabetes prevalence in SA is currently underway for the second National Burden of Disease Study, which should improve estimation of the denominator (population in need of treatment).[4]

For the purpose of our analysis, a modelled estimate of diabetes prevalence and treatment coverage per district and per year was instead generated for UHC10.

A machine learning algorithm was trained with data from SADHS 2016, which includes biomarkers allowing for a direct estimation of diabetes status, to predict individual probabilities of being diabetic from demographic (age, sex, ethnicity) and bio-behavioural (body mass index, waist circumference, current smoking) characteristics and self-reported previous diagnosis and use of medication.

To improve accuracy the model employs a combination of classification algorithms (namely Random Forest, SVM, Recursive Partitioning, Boosted Regression Model) and calculates the probabilities of being diabetics based on the set of predictors listed above as a weighted average of the probabilities predicted by the individual models, the weights being the estimated accuracy of each prediction (calculated by cross-validation).

Table A1 shows the estimates accuracy of the individual models.

Table A1: Accuracy of the four ML models used to predict diabetes status based on demographic and bio-behavioural predictors. Cross-validation estimates and 95% Confidence Intervals.

		95% Confidence Interval	
**Model**	**Accuracy [%]**	**lb**	**ub**
Random Forest	80.2	66.7	90.0
SVM	76.5	61.1	86.7
Recursive Partitioning	77.7	63.3	86.7
Boosted Regression Model	80.6	66.7	90.0

lb, ub = upper and lower bounds of the 95% confidence interval.

Sensitivity and specificity of the overall procedure was also estimated by cross-validation. The results are shown in Table A2.

Table A2: Sensitivity and specificity ML models used to predict diabetes status based on demographic and bio-behavioural predictors. Cross-validation estimates and 95% Confidence Intervals.

	**Sensitivity [%]**			**Specificity [%]**		
	**estimate**	**lb**	**ub**	**estimate**	**lb**	ub
Random Forest	60.7	30.0	80.0	90.0	80.0	100.0
SVM	60.3	30.0	80.0	84.6	70.0	100.0
Recursive Partitioning	50.0	20.0	70.0	91.5	80.0	100.0
Boosted Regression Model	61.5	30.0	80.0	90.2	80.0	100.0

lb, ub = upper and lower bounds of the 95% confidence interval.

The model was applied by using as predictors demographic and bio-behavioural characteristics of the individuals sampled in the five waves of the NiDS survey to predict the individual probability of being diabetic. The predict probabilities were averaged taking into account the complex sampling scheme of each wave and the result considered an estimate of the prevalence of diabetes in the population for the respective wave.

To correct for the imperfect sensitivity and specificity of the predictive mode, the estimated prevalences were corrected with the formula:

$$p = \frac{\hat{p} + (Sp - 1)}{Se + (Sp - 1)}$$

The performance of the procedure was assessed by using random subsets of the SADHS datasets with different proportions of diabetics and comparing the model-predicted prevalences with the observed ones. An example result is shown in Figure A6.

Figure A6: Observed vs. model-predicted prevalences of diabetes in random subsets of the SADHS dataset.

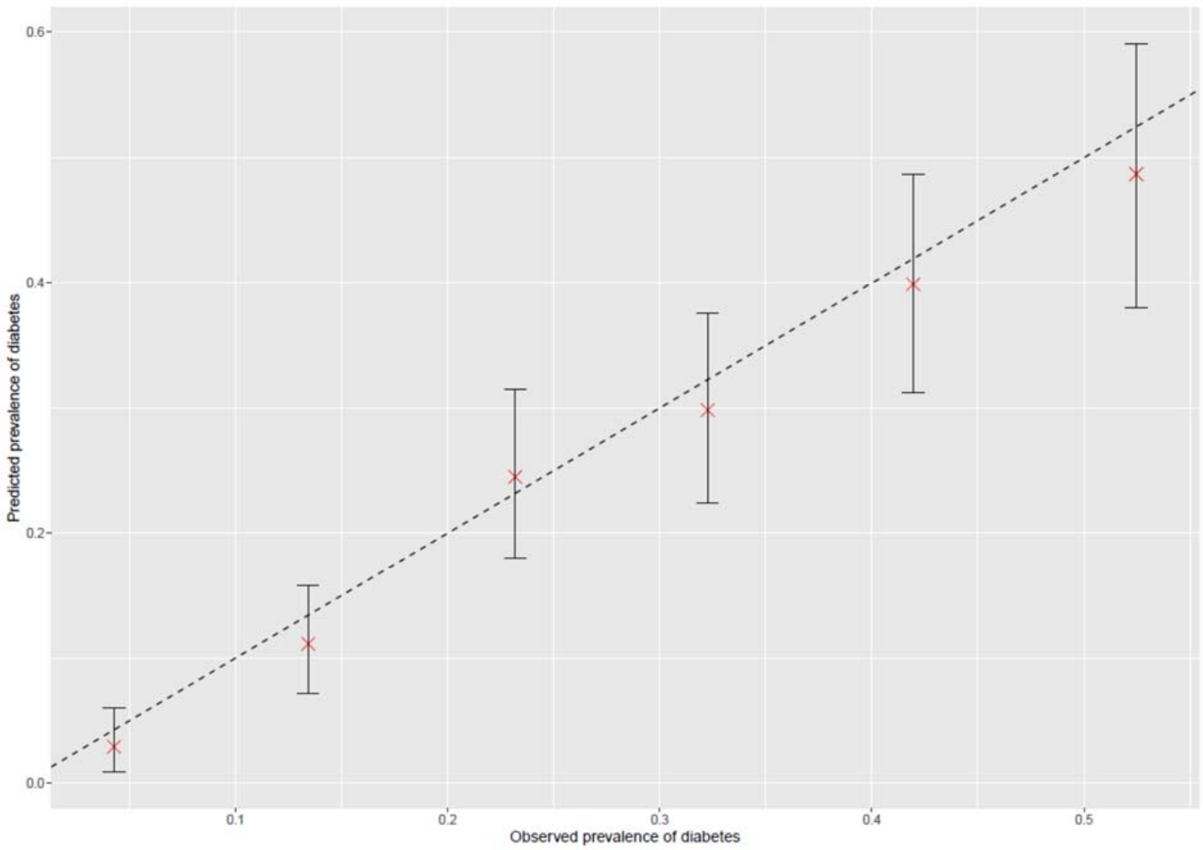

X = model-predicted prevalence of diabetes (and 95% Confidence Intervals).

The proportion of subjects on medication was directly estimated from self-reported data, and treatment coverage was calculated as the ratio between population proportion of treated and diabetes prevalence. A smooth variation over time was assumed for treatment coverage, and final annual estimates were obtained by thin-plate spline smoothing.

1.5. Estimation of proportion of population not covered by medical insurance

The proportion of the insured population per district was estimated by Insight Actuaries [https://www.insight.co.za/] using a small area model based on Census 2011, Community Survey 2016 and scaled using the General Household Survey 2018 and Council for Medical Schemes data. The predictors included gender of the household head, age of the household head, province, residence in a metropolitan municipality, income category, and number of household members. This estimate of medical schemes coverage was then used with the population time series in DHIS to calculate the uninsured population.[5, 6]

2. Additional Results

Table A3: UHC SCI by national and provincial level, 2007-08 and 2016-17

			Country		Province																	
			SA		EC		FS		GP		KZ		LP		MP		NC		NW		WC	
		Indicator	2007-2008	2016-2017	2007 & 2008	2016 & 2017	2007 & 2008	2016 & 2017	2007 & 2008	2016 & 2017	2007 & 2008	2016 & 2017	2007 & 2008	2016 & 2017	2007 & 2008	2016 & 2017	2007 & 2008	2016 & 2017	2007 & 2008	2016 & 2017	2007 & 2008	2016 & 2017
RMNCH	1	Couple year protection rate	31	70	31	74	34	67	24	60	25	74	36	85	28	71	32	60	24	60	61	81
	2	Antenatal 1st visit coverage before 20 weeks	29	51	19	38	40	49	24	53	29	51	36	54	29	66	46	63	29	52	41	55
	3	Immunisation under 1 year coverage	75	77	65	68	95	71	85	77	64	81	80	70	71	90	88	91	68	69	96	81
	4	Pneumonia case fatality under 5 years rate (smoothed	77	94	70	89	70	93	79	93	82	93	69	91	65	91	84	94	70	91	98	99
Infectious	5	Tuberculosis effective treatment coverage	51	56	51	57	52	55	54	57	48	56	46	55	49	56	54	52	41	54	57	55
	6	Antiretroviral effective coverage		32		30		37		25		36		39		38		27		26		34
	8	Percentage of households with access to improved sa	64	76	45	74	68	79	87	89	57	65	28	52	55	60	81	79	51	65	93	94
NCDs	9	Age-standardised prevalence of non-raised blood pres	48	60	44	61	47	54	50	63	48	57	61	67	45	70	39	50	39	61	41	53
	10	Diabetes treatment coverage	44	37	47	36	41	33	50	38	55	35	34	33	39	40	52	47	58	45	71	34
	11	Cervical cancer screening coverage	43	64	31	64	39	55	41	52	51	91	49	57	36	78	29	43	51	70	47	58
	12	Tobacco non-smoking prevalence	79	81	83	83	76	80	79	79	83	87	85	88	83	82	65	67	79	80	63	69
Capacity	13	Hospital beds per 10 000 target population (rescaled)	100	100	100	100	100	100	100	100	100	100	96	81	86	73	100	100	98	80	100	100
	14	Health worker density (rescaled)	10	15	8	15	10	13	9	15	11	15	9	15	8	12	11	19	8	12	13	16
	15	Proportion of health facilities with essential medicines	64	82	51	83	47	65	90	87	100	88	51	58	76	85	54	88	100	98	1	96
	16	Environmental health services compliance rate		63		62		76		71		59		55		64		57		66		63
		**RMNCH**	48	71	40	64	55	68	44	69	44	73	51	74	44	79	57	75	42	67	70	77
		**Infectious**	57	51	48	50	59	54	68	50	52	51	36	48	52	51	66	48	46	45	72	56
		**NCDs**	52	58	48	59	49	53	53	56	58	63	54	58	48	65	44	51	55	63	54	52
		**Capacity**	40	50	34	49	36	44	43	51	48	51	36	41	37	43	39	55	42	45	11	54
		**UHC Index**	46	57	40	55	46	54	47	56	50	59	46	54	43	58	46	56	46	54	35	59

Figure A7: Range of UHC SCI and component indicators at national, provincial and district level, 2016-17

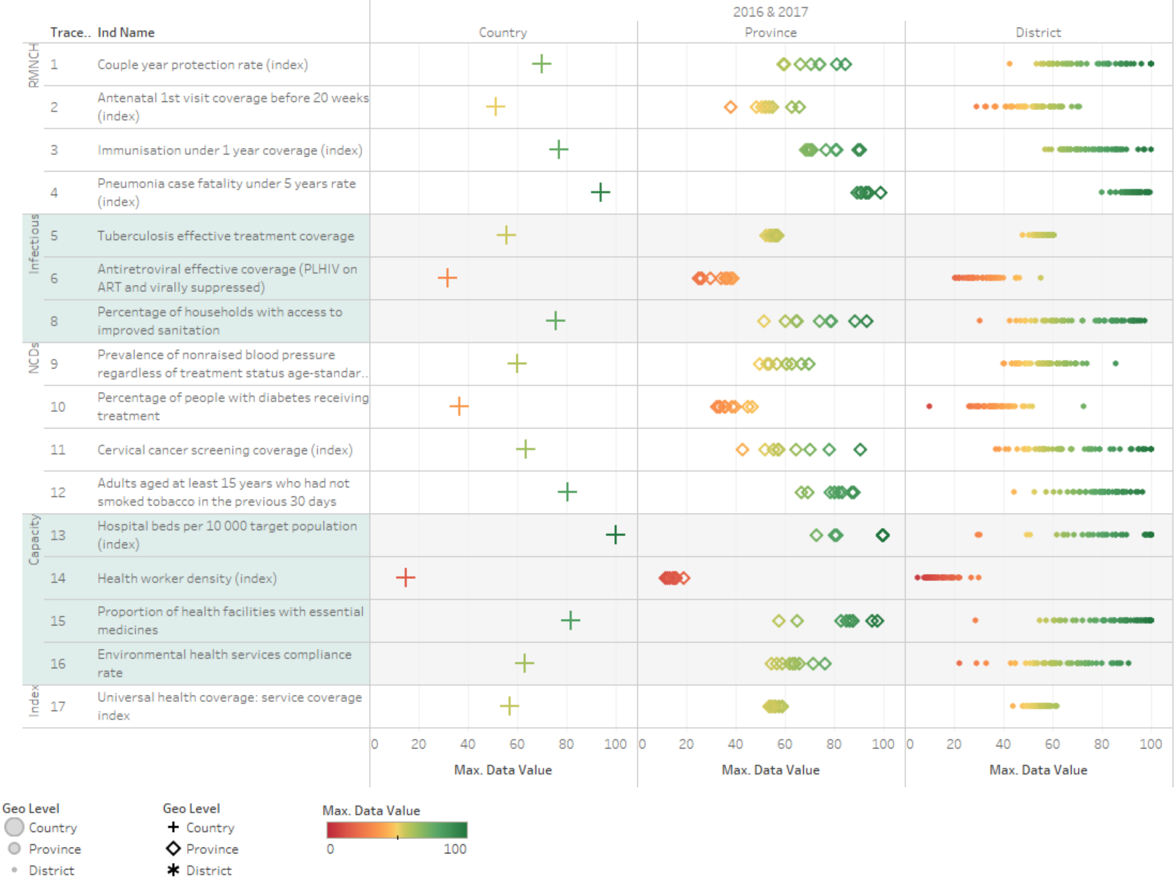

References

1. Stokes A, Berry KM, McHiza Z, Parker WA, Labadarios D, Chola L, et al. Prevalence and unmet need for diabetes care across the care continuum in a national sample of South African adults: Evidence from the SANHANES-1, 2011-2012. PloS One. 2017;12(10):e0184264. Epub 2017/10/03.
2. Day C, Groenewald P, Laubscher R, Chaudhry S, Van Schaik N, Bradshaw D. Monitoring of non-communicable diseases such as hypertension in South Africa: challenges for the post-2015 global development agenda. S Afr Med J. 2014;104(10):680-7. Epub 2014/11/05.
3. Cois A. 11. Non-communicable diseases. In: Massyn N, Peer N, Padarath A, Day C, editors, editors. District Health Barometer 2016/17. Durban: Health Systems Trust; 2017.
4. Pheiffer C, Pillay-van Wyk V, Joubert JD, Levitt N, Nglazi MD, Bradshaw D. The prevalence of type 2 diabetes in South Africa: a systematic review protocol. BMJ Open. 2018;8(7):e021029. Epub 2018/07/13.
5. Massyn N, Pillay Y, Padarath A, editors. District Health Barometer 2017/18. Durban: Health Systems Trust; 2018.
6. Massyn N, Barron P, Day C, Ndlovu N, Padarath A, editors. District Health Barometer 2018/19. Durban: Health Systems Trust; 2020.
